# Redox potential changes during ATP‐dependent corrinoid reduction determined by redox titrations with europium(II)–DTPA

**DOI:** 10.1002/pro.3699

**Published:** 2019-08-07

**Authors:** Hendrike Dürichen, Gabriele Diekert, Sandra Studenik

**Affiliations:** ^1^ Institute of Microbiology, Department of Applied and Ecological Microbiology Friedrich Schiller University Jena Jena Germany

**Keywords:** ATP‐dependent corrinoid reduction, corrinoid cofactor, europium, *O*‐demethylase, potentiometric titration, thermodynamically unfavorable electron transfer

## Abstract

Corrinoids are essential cofactors of enzymes involved in the C_1_ metabolism of anaerobes. The active, super‐reduced [Co^I^] state of the corrinoid cofactor is highly sensitive to autoxidation. In *O*‐demethylases, the oxidation to inactive [Co^II^] is reversed by an ATP‐dependent electron transfer catalyzed by the activating enzyme (AE). The redox potential changes of the corrinoid cofactor, which occur during this reaction, were studied by potentiometric titration coupled to UV/visible spectroscopy. By applying europium(II)–diethylenetriaminepentaacetic acid (DTPA) as a reductant, we were able to determine the midpoint potential of the [Co^II^]/[Co^I^] couple of the protein‐bound corrinoid cofactor in the absence and presence of AE and/or ATP. The data revealed that the transfer of electrons from a physiological donor to the corrinoid as the electron‐accepting site is achieved by increasing the potential of the corrinoid cofactor from −530 ± 15 mV to −250 ± 10 mV (*E*_SHE_, pH 7.5). The first 50 to 100 mV of the shift of the redox potential seem to be caused by the interaction of nucleotide‐bound AE with the corrinoid protein or its cofactor. The remaining 150–200 mV had to be overcome by the chemical energy of ATP hydrolysis. The experiments revealed that Eu(II)–DTPA, which was already known as a powerful reducing agent, is a suitable electron donor for titration experiments of low‐potential redox centers. Furthermore, the results of this study will contribute to the understanding of thermodynamically unfavorable electron transfer processes driven by the power of ATP hydrolysis.

## 1 INTRODUCTION

Protein‐bound corrinoid cofactors play an essential role as methyl group carriers in the C_1_ metabolism of anaerobes.^1^ To bind methyl groups, the corrinoid cofactor has to be in its super‐reduced [Co^I^] state, which is highly sensitive to autoxidation. To maintain the catalytic reaction cycle, inadvertently oxidized corrinoid cofactors ([Co^II^] state) have to be re‐reduced to [Co^I^]. This activation reaction is challenging since the midpoint potential of the [Co^II^]/[Co^I^] couple^2^, ^3^ is lower than the redox potentials of the most negative physiological electron donors ferredoxin and flavodoxin.^4^ In the acetogen *Acetobacterium dehalogenans*, protein‐bound corrinoid cofactors are part of the ether‐bond‐cleaving *O*‐demethylases. These four‐protein component enzyme systems catalyze the transfer of substrate methyl groups to tetrahydrofolate, which is the key reaction in the methylotrophic metabolism of these strictly anaerobic bacteria.^5^, ^6^ In *A. dehalogenans*, the reduction of inadvertently oxidized corrinoid cofactors involved in *O*‐demethylation is achieved in an ATP‐dependent reaction catalyzed by the activating enzyme (AE), which harbors a [2Fe‐2S] cluster.^7^ During this reaction, the midpoint potential of the [Co^II^]/[Co^I^] couple is increased by at least 200 mV to about −300 mV, which makes the electron transfer from the reduced Fe/S cluster of AE (*E*_SHE_ = −330 mV; pH 7.5) to the protein‐bound corrinoid cofactor feasible.^3^, ^7^ The precise shift of the potential during this reaction was unknown so far, since in the absence of AE and ATP, the reductant titanium(III) citrate is not strong enough to fully reduce the protein‐bound corrinoid cofactors involved in *O*‐demethylation. The lowest potential reached with Ti(III) citrate was about −550 mV and did not result in significant [Co^I^] formation.^7^

Also other types of enzymes, such as nitrogenase, 2‐hydroxyacyl‐CoA dehydratases, and benzoyl‐CoA reductases use the power of ATP hydrolysis to enable thermodynamically unfavorable electron transfer processes, thus maintaining the catalytic reaction cycle of the enzymes.^8^, ^9^ The activator, which transfers the electron from a donor to the catalytic subunit, is in all known cases a Fe/S cluster‐containing protein, which binds and hydrolyzes ATP. In nitrogenase, 2‐hydroxyacyl‐CoA dehydratases, and benzoyl‐CoA reductases, the activator is composed of two subunits (homodimer or heterodimer) with a bridging [4Fe–4S] cluster.^9^, ^10^ The monomeric activator of corrinoid‐dependent methyltransferases (like *O*‐demethylases or the methyltransferases of the Wood–Ljungdahl pathway) contains one [2Fe–2S] or two [4Fe–4S] clusters.^7^, ^11^, ^12^ Although a few structures of the activators, either as single proteins or in complex with its protein substrate, were solved (e.g., 13, 14, 15, 16, 17), the mechanisms of ATP‐dependent activation are not yet fully understood; however, they seem to differ among the enzymes. In benzoyl‐CoA reductases and 2‐hydroxyacyl‐CoA dehydratases, the hydrolysis of ATP induces a decrease in the potential of the donated electrons from around −400 mV to ca. –600 mV or −800 mV, respectively, allowing its transfer to the active site.^9^ In *O*‐demethylases, the midpoint potential of the corrinoid cofactor ([Co^II^]/[Co^I^]), which is the electron‐accepting site, is increased during activation from <−500 mV to about −300 mV allowing the electron flow from a low potential donor to the more positive [Co^II^]–corrinoid cofactor.^3^, ^7^ In nitrogenase and during activation of the corrinoid iron–sulfur protein (CoFeSP), the main function of ATP hydrolysis seems to be the dissociation of a protein complex composed of the activator and the electron‐accepting protein, which enables the unidirectional flow of electrons.^17^, ^18^ How ATP binding and/or hydrolysis in combination with conformational changes of the participating proteins accomplish these tasks is still under investigation.

In this study, for the first time, we were able to determine the midpoint potential of the [Co^II^]/[Co^I^] couple of the nonactivated protein‐bound corrinoid cofactor of *O*‐demethylase enzyme systems. The new findings allow predictions of the redox potential shifts, which occur during ATP‐dependent corrinoid reduction catalyzed by AE. To achieve complete corrinoid reduction, Ti(III) citrate was replaced by the more powerful reducing agent europium(II)–DTPA, which was used previously for the reduction of iron‐containing redox centers.^19^, ^20^, ^21^ To the best of our knowledge, this is the first report on applying Eu(II)–DTPA for the determination of redox potentials of protein‐bound cofactors.

## 2 RESULTS AND DISCUSSION

**Figure 1:**
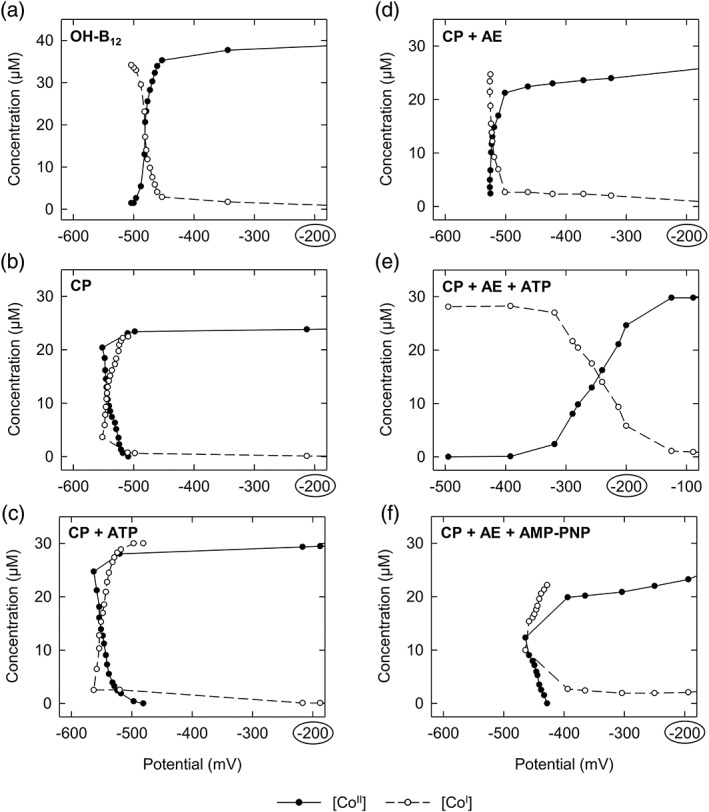
Redox titration curves of free and protein‐bound corrinoid cofactor. Eu(II)–DTPA was used for the stepwise reduction of the [Co^II^] (filled circles) to the [Co^I^] form (open circles), which was detected spectrophotometrically at 475 and 386 nm, respectively. (a) Hydroxocobalamin, (b) protein‐bound corrinoid cofactor, (c) protein‐bound corrinoid cofactor in the presence of 2 mM ATP, (d) protein‐bound corrinoid cofactor in the presence of AE, (e) protein‐bound corrinoid cofactor in the presence of AE and 2 mM ATP, (f) protein‐bound corrinoid cofactor in the presence of AE and 2 mM AMP–PNP. AE was applied in half‐molar concentration to that of CP. AE, activating enzyme; CP, corrinoid protein; OH‐B_12_, hydroxocobalamin

To shed light on the reaction mechanism of ATP‐dependent corrinoid reduction, it is of importance to analyze the redox potential differences, which emerge during this reaction. So far, Ti(III) citrate was applied for the redox titrations of the protein‐bound corrinoid cofactors of *O*‐demethylases. However, this compound was not strong enough to reduce [Co^II^] to [Co^I^] in the absence of AE and ATP. In addition, increasing concentrations of Ti(III) citrate interfered with the applied assay.^3^ In the current study, Ti(III) citrate was replaced by Eu(II)–DTPA and was added stepwise to the protein solutions. The redox potential was measured and, concomitantly, UV/visible absorption spectra were recorded. Midpoint potentials ([Co^II^]/[Co^I^] transition) were determined for the protein‐bound corrinoid cofactor (i) without additives, (ii) in the presence of ATP, (iii) in the presence of AE, (iv) in the presence of AE and ATP, and (v) in the presence of AE and AMP‐PNP (a non‐hydrolysable analogue of ATP), respectively. For comparison, the midpoint potential of hydroxocobalamin (free cofactor) was determined by the same method. Representative titration curves are shown in Figure 1. The calculated midpoint potentials are summarized in Table 1. For complete reduction of the corrinoid cofactor, 4–10 μL of freshly prepared 0.4 M Eu(II)–DTPA had to be added to the cuvettes. This corresponds to a final concentration of about 1–2.5 mM. The pH remained stable during titration. The midpoint potential of the nonactivated protein‐bound corrinoid cofactor (Figure 1b), which was determined for the first time, was −530 ± 15 mV (*E*_SHE_, pH 7.5). During the titration process, a certain potential had to be overcome until [Co^II^] reduction started. Beyond this potential and without further addition of Eu(II)–DTPA (“point of no return”), [Co^II^] was completely reduced to [Co^I^] along with a slight increase of the redox potential. This increase was not observed during titration of the free cofactor (Figure 1a) and cannot be explained so far. Possibly, it is caused by conformational changes of the protein environment, which might occur in the course of the reduction process. Re‐oxidation and re‐reduction of the protein‐bound corrinoid cofactor was possible (data not shown), demonstrating the reversibility of the titration process and indicating that the procedure was nondestructive. The addition of ATP to the titration mixture did not obviously affect the redox potential of the [Co^II^]/[Co^I^] couple and the shape of the titration curve (Figure 1c). When AE was present (in the absence of ATP), the reduction of protein‐bound [Co^II^] to [Co^I^] also occurred after reaching a “point of no return” without further addition of Eu(II)–DTPA (Figure 1d). The shape of the titration curve changed to a normal pattern as observed for the free cofactor. The midpoint potential of the [Co^II^]/[Co^I^] couple in the presence of AE was −500 ± 20 mV (*E*_SHE_, pH 7.5), which is close to the value determined without additive (Figure 1b). In contrast, the binding of the activator RACo to its corrinoid substrate CoFeSP caused a markedly decrease of the midpoint potential of the [Co^II^]/[Co^I^] couple of CoFeSP. The potential changed from −450 mV (CoFeSP alone) to below −600 mV (+ RACo), resulting in a stabilization of the [Co^II^] form.^17^ This might explain why the predicted function of ATP hydrolysis during corrinoid reduction differs between *O*‐demethylases and CoFeSP.

**Table 1:** Midpoint potential (*E*_SHE_, pH 7.5) of free and protein‐bound corrinoid cofactor

Sample	Midpoint potential (mV)
OH‐B_12_	−460 ± 15
CP	−530 ± 15
CP + ATP	−525 ± 25
CP + AE	−500 ± 20
CP + AE + ATP	−250 ± 10
CP + AE + AMP‐PNP	−450 ± 10

*Note*: Redox titrations were performed via potentiometric titration coupled to UV/visible spectroscopy. Eu(II)–DTPA was applied as the reductant. Representative titration curves are shown in Figure 1.

Abbreviations: AE, activating enzyme; CP, corrinoid protein; OH‐B_12_, hydroxocobalamin.

**Figure 2:**
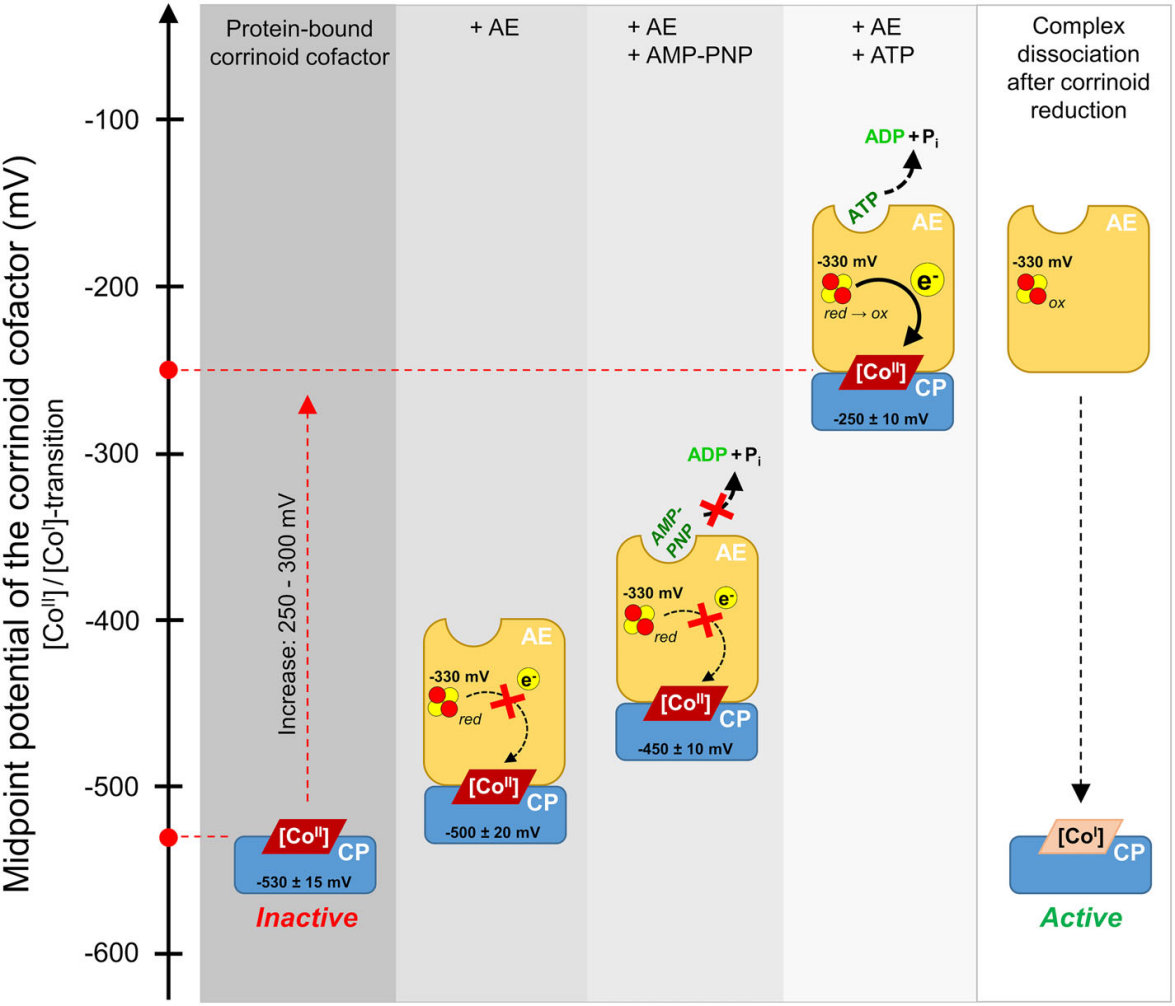
Proposed re‐activation cycle of protein‐bound corrinoid cofactors involved in *O*‐demethylation. The position of the corrinoid cofactor (illustrated as rhomboid) represents its midpoint potential. The Fe/S cluster of the activating enzyme is depicted in red and yellow circles. AE, activating enzyme; CP, corrinoid protein, [Co] = corrinoid cofactor in the respective oxidation state

The titration of the protein‐bound corrinoid cofactor in the presence of AE and ATP, which was also successfully performed earlier with Ti(III) citrate as reductant,^3^, ^7^ led to a noticeable increase of the midpoint potential of the [Co^II^]/[Co^I^] couple from −530 ± 15 mV (without additives; Figure 1b) to −250 ± 10 mV (Figure 1e). Based on these data, it is now possible to quantify the potential increase, namely 250–300 mV, which is achieved during ATP‐dependent corrinoid reduction. To study the effect of nucleotide binding, and not hydrolysis, on the midpoint potential of the protein‐bound corrinoid cofactor, ATP was exchanged by the nonhydrolysable ATP‐analogue AMP–PNP in the titration mixture (Figure 1f). Under these conditions, the midpoint potential of the [Co^II^]/[Co^I^]‐couple was −450 ± 10 mV. This result indicates that the interplay of nucleotide‐bound AE with the corrinoid protein or its cofactor already induces a positive shift of the potential by 50–100 mV. The remaining 150–200 mV had to be overcome by the chemical energy of ATP hydrolysis. In theory, the hydrolysis of one ATP (Δ*G*′ ≈ 50 kJ/mol) provides enough energy to overcome a redox barrier of 300 mV considering a one‐electron transfer and an efficiency of 60%. Accordingly, a stoichiometry of one ATP hydrolyzed per electron transferred was determined previously.^22^ Finally, the increase of the midpoint potential from −530 to −250 mV, which is caused by AE in the presence of ATP, enables the transfer of electrons from a physiological donor (e.g., ferredoxin or flavodoxin) via the Fe/S cluster of AE (*E*_SHE_ = −330 mV; pH 7.5) to the protein‐bound corrinoid cofactor in the [Co^II^] state (Figure 2). The stoichiometry of ATP hydrolyzed per electron transferred differs among the known activation systems. Whereas in nitrogenase and most probably also in 2‐hydroxyacyl‐CoA dehydratases, two ATPs have to be invested for the transfer of one electron,^23^, ^24^, ^25^ in benzoyl‐CoA reductases and *O*‐demethylases one ATP per electron is sufficient.^22^, ^26^, ^27^ The latter stoichiometry should also be true for RACo and CoFeSP, but has to be proven. In addition, differences occur among the enzymes to be activated regarding the need of ATP per catalytic reaction cycle. Nitrogenase and benzoyl‐CoA reductases rely on ATP for the transfer of electrons in each catalytic cycle,^8^, ^27^ whereas the reactions catalyzed by 2‐hydroxyacyl‐CoA dehydratases and corrinoid‐dependent methyltransferases may run for several 100 cycles until reactivation is required.^25^, ^28^, ^29^

The midpoint potential of the [Co^II^]/[Co^I^] couple of hydroxocobalamin, which served as reference, was determined to be −460 mV ± 15 mV (*E*_SHE_, pH 7.5) (Figure 1a). A similar result was obtained when Eu(II)–DTPA was replaced by Ti(III) citrate (data not shown). This value is in accordance with the previously reported standard redox potential of base‐off hydroxocobalamin (−490 mV).^30^ However, under the conditions applied here (pH 7.5), a base‐on form should exist. Its standard redox potential was earlier determined to be near −600 mV.^30^ This value differs more than 100 mV from our result and might be explained by the different methods applied: Lexa and Saveant used cyclic voltammetry,^30^ whereas our value originates from potentiometric titration experiments.

## 3 CONCLUSIONS

Eu(II)–DTPA was identified as suitable electron donor for titration experiments of low‐potential redox centers. It extends the small number of reductants known to be applicable for the generation of [Co^I^] corrinoids.^31^ By the application of Eu(II)–DTPA, we identified the redox potential differences, which occur during the reduction of protein‐bound corrinoid cofactors involved in *O*‐demethylation. In contrast to other thermodynamically unfavorable electron transfer processes, in which the potential of the electron is lowered to allow its transfer to the active site,^9^ in *O*‐demethylases the potential of the electron‐accepting site is increased to enable a spontaneous flow of electrons (Figure 2). The investment of one ATP is sufficient to overcome a redox barrier of approximately 300 mV.

## 4 MATERIALS AND METHODS

### 4.1 Chemicals and gases

Chemicals were purchased in the highest available purity from Carl Roth GmbH (Karlsruhe, Germany), GERBU Biotechnik GmbH (Heidelberg, Germany), IBA GmbH (Göttingen, Germany), Roche Diagnostics GmbH (Mannheim, Germany), and Sigma‐Aldrich Chemie GmbH (Taufkirchen, Germany). Nitrogen (purity 5.0) and forming gas (95% N_2_, 5% H_2_) were supplied by Linde AG (Pullach, Germany).

### 4.2 Production and purification of the recombinant proteins

The activating enzyme (AE; Accession number: WP_026395886) and corrinoid protein (CP; Accession number: WP_026394334) of the vanillate *O*‐demethylase of *A. dehalogenans* were heterologously produced as C‐terminal *Strep*‐Tag® fusions in *Escherichia coli* BL21 (DE3) as described previously.^7^, ^22^ Protein purification of AE was performed in the presence of oxygen by affinity chromatography on *Strep*‐Tactin® according to the manufacturer's protocol (IBA GmbH, Göttingen, Germany) using 65 mM sodium phosphate buffer pH 8.5 containing 150 mM NaCl as basic buffer. Under anoxic conditions, the Fe/S clusters of purified AE were reconstituted by adding 5 mM DTT and a fivefold molar excess (with respect to the iron and sulfur content of AE) of ammonium iron(III) citrate and lithium sulfide. After incubation for at least 24 hr at 8°C, the protein sample was concentrated by ultrafiltration (Vivaspin 20, 10 kDa cut‐off; Sartorius AG, Göttingen, Germany) at 4,000*g* and 10°C. To remove unbound iron and sulfur, AE was washed with 65 mM sodium phosphate buffer at pH 7.5 containing 150 mM NaCl. The purification of CP was done according to Schilhabel et al. with some modifications.^7^ After the Q Sepharose column, an additional chromatography step (hydrophobic interaction) was included to further enrich the CP apoprotein. After Q Sepharose, the CP‐containing fractions were pooled and ammonium sulfate was added to a final concentration of 0.8 M. The sample was passed through a Phenyl Superpose column (10/10) equilibrated with 50 mM Tris–HCl at pH 7.5 containing 0.5 mM DTT (basic buffer) and 0.8 M ammonium sulfate at a flow rate of 0.5 mL min^−1^. Separation of proteins was achieved by a linear decreasing gradient of ammonium sulfate from 0.8 to 0 M in basic buffer within five column volumes and at a flow rate of 1 mL min^−1^. The reconstitution of CP with hydroxocobalamin was done as described previously with an increased concentration of DTT (20 mM) and an incubation time of at least 48 hr at 8°C.^7^ After Mono Q chromatography, the fractions containing holo‐CP were pooled and concentrated via ultrafiltration (see above). The corrinoid content of the protein was calculated from UV/visible spectra using Δ*ε*_386nm_ = 21 mM^−1^ cm^−1^ for [Co^I^] and *ε*_475nm_ = 9.2 mM^−1^ cm^−1^ for [Co^II^].^3^, ^32^

### 4.3 Analytical methods

Protein determination was performed according to the method of Bradford with bovine serum albumin as the standard.^33^

### 4.4 Determination of the corrinoid reduction activity

The corrinoid reduction activity was determined spectrophotometrically under anoxic conditions in a final volume of 100 μL as described previously.^7^ The assay mixture further contained 250 mM potassium acetate. The activity was calculated from the kinetics of [Co^I^] formation at 386 nm (Δ*ε*_386nm_ = 21 mM^−1^ cm^−1^).^3^

### 4.5 Determination of the midpoint potential of the corrinoid cofactor

The midpoint potential of the protein‐bound corrinoid cofactor was determined by potentiometry coupled to UV/visible absorption spectroscopy under strictly anaerobic conditions at 18°C. Eu(II)–DTPA was used as the electron donor. For preparation of a 0.4 M stock solution, 90 mg of Eu(II) chloride were dissolved in 1 mL of 0.4 M DTPA at pH 8.5. Stock solutions of ATP and AMP–PNP (20 mM each) were prepared in 125 mM Tris–HCl at pH 7.5 containing 100 mM MgCl_2_. The titration was carried out in 2‐mL quartz cuvettes filled with 1.5 mL of the sample mixture. The final concentrations of CP, AE, and ATP/AMP‐PNP were as follows: 30 μM, 15 μM, and 2 mM, respectively. Tris–HCl of 50 mM at pH 7.5 was used as the buffer. Eu(II)–DTPA was added stepwise to the sample. After the equilibrium was reached, the UV/visible absorption spectrum was recorded and the redox potential of the solution was measured concomitantly with an Ag/AgCl electrode (Pt 5900 A, SI Analytics GmbH, Mainz, Germany). For calculation of the potential versus the standard hydrogen electrode (SHE), 207 mV had to be added to the recorded potential. The calculation of the midpoint potential of the protein‐bound corrinoid cofactor occurred with the Nernst formula. The dilution caused by the addition of Eu(II)–DTPA was taken into account for the calculations. At least two measurements were performed per condition. At the start and at the end of the titration experiment, the pH was measured to control and ensure its stability.

## CONFLICT OF INTEREST

The authors declare no potential conflict of interest.
